# Characteristics differ based on usual cigar-type use among U.S. adults: Analysis from the tobacco use supplement to the current population survey

**DOI:** 10.1016/j.pmedr.2021.101560

**Published:** 2021-09-15

**Authors:** Sunday Azagba, Jessica L. King, Lingpeng Shan

**Affiliations:** a Ross and Carol Nese College of Nursing, The Pennsylvania State University, University Park, PA 16802, USA; b Department of Health & Kinesiology, University of Utah, Salt Lake City, UT, USA; c Division of Biostatistics, College of Public Health, The Ohio State University, Columbus, OH 43210, USA

**Keywords:** Cigar choices, Cigar-type preferences, Large cigars, Cigarillos, Little filtered cigars

## Abstract

The tobacco products landscape is continually shifting, and there are concerns about the increased popularity of non-cigarette tobacco products, including cigars. This study examines characteristics associated with usual cigar-type use. Data are from the 2018–19 Tobacco Use Supplement to the Current Population Survey. Multinomial logistic regression was used to assess the association between sociodemographic characteristics and cigar-type use (i.e., large cigars, cigarillos, and little filtered cigars). Analyses also examined factors relative to large cigar use and further stratified by sex. Of 137,221 adults included in the study, 1467 used large cigars most often, 513 used cigarillos most often, 446 used little filtered cigars most often, and the remaining 134,795 did not use cigars. In adjusted models, males had greater odds for using all types of cigars relative to non-use. In contrast, males were less likely to use cigarillos (AOR 0.28, 95% CI 0.20–0.41) and filtered cigars (AOR 0.20, 95% CI 0.14–0.28) relative to large cigars. Black adults had greater odds of using all types of cigars relative to non-use, and cigarillos (AOR 3.55, 95% CI 2.47–5.08) and filtered cigars (AOR 2.50, 95% CI 1.70–3.68) relative to large cigars. Education, income, and other tobacco use also varied according to cigar type. Characteristics of those who usually use large cigars differed significantly from those who usually use cigarillos, little filtered cigars, or reported no cigar use.

## Highlights

• Significant demographic heterogeneity was found based on usual cigar type.
• Males were more likely to use all types of cigars relative to non-use.
• Males were less likely to use cigarillos and filtered cigars relative to large cigars.
• Black adults had greater odds for using all types of cigars relative to non-use.
• Education, income, and other tobacco use also varied according to cigar type.

## 1 Introduction

Significant progress has been made in reducing cigarette smoking over the past few decades (U.S. Department of Health and Human Services, 2014); however, there are concerns about the increased popularity of other tobacco products, including cigars. For example, cigar consumption has risen by 85%, from 6.2 billion cigars smoked in 2000 to over 11 billion in 2015. Cigarette consumption decreased by nearly 40%, from 435 billion to 267 billion during the same period (Wang, 2016). Cigar smoking has become a public health burden in the U.S. Recent estimates indicate 3.6%, or 8.7 million, U.S. adults smoked cigars some days or every day in 2019 (Cornelius et al., 2020). Cigars are not a safe alternative to cigarettes. It is well documented that cigar smoke contains many of the same toxic and carcinogenic compounds as traditional cigarette smoke (National Cancer Institute, 1998). Cigar use is associated with an increased risk of lung, oral, esophageal, and laryngeal cancers and coronary heart disease (Cornelius et al., 2020). A previous study estimated that in 2010 alone, regular cigar smoking was responsible for approximately 9000 premature deaths and economic costs of 23 billion dollars (Nonnemaker et al., 2014).

In the U.S., the three commonly sold cigar types are large cigars, cigarillos, and little filtered cigars (National Cancer Institute, 1998). The cigar types differ in size and production process: large cigars typically contain at least one-half ounce of aged, fermented tobacco (i.e., as much as a pack of cigarettes) and usually take 1–2 h to smoke; cigarillos tend to be between 3 and 4 in., contain about 3 g of tobacco, and typically exclude a filter; and little filtered cigars are about the same size and shape as cigarettes and are often used interchangeably (National Cancer Institute, 1998, Maxwell, 2015). Some common brands for large cigars and cigarillos are Black and Milds, Swisher Sweets, Phillies, and Prime time; Winchester and Cheyenne are common brands for little filtered cigars. Some studies have identified varying characteristics associated with cigar use, including sociodemographic factors and co-use with other substances (Corey et al., 2018, Borawski et al., 2010, Chen-Sankey et al., 2021, Richardson et al., 2013, Cohn et al., 2015). A study using Wave 3 of the Population Assessment of Tobacco and Health Study found that non-Hispanic Black adults were more likely to smoke cigars in the past 30 days, with results consistent across cigar types (Chen-Sankey et al., 2021). Other national studies have identified some key demographic differences based on the usual cigar type. Adults who use large cigars are more likely to be non-Hispanic White, male, older, and report higher income and educational attainment (Corey et al., 2018, Borawski et al., 2010, Richardson et al., 2013, Corey et al., 2014). In contrast, adults who report using cigarillos or filtered cigars are more likely to be younger, non-Hispanic Black, and have lower income and educational attainment (Corey et al., 2018, Borawski et al., 2010, Richardson et al., 2013, Corey et al., 2014). Previous studies report high use of cigarettes among this population (Corey et al., 2014), and that cigarette use is less common among those who use large cigars compared to those using cigarillos or filtered cigars (Corey et al., 2018, Richardson et al., 2013). While the tobacco products landscape has witnessed significant changes in the last few years with the emergence of new products, research on usual cigar-type use has been limited. The current study examined characteristics associated with usual cigar-type use (large cigars, cigarillos, and little filtered cigars) using a nationally representative U.S. sample of adults from the 2018–19 Tobacco Use Supplement to the Current Population Survey (TUS-CPS) study.

## 2 Methods

### 2.1 Data

The Tobacco Use Supplement to the Current Population Survey (TUS-CPS) is a large household survey among the civilian noninstitutionalized population 16 years of age and older in the United States. It is administered by the Census Bureau and sponsored by the National Cancer Institute (NCI). The CPS is a monthly labor force survey conducted in more than 50,000 interviewed households across the country. Since 1992, the TUS-CPS has been conducted every three to four years as a supplement of the CPS to assess many topics, including smoking status, amount smoked, smoking history, quit attempts, intention to quit, level of nicotine dependence, and other tobacco-related topics. We excluded 234 respondents with “No response,” “Refused,” or “Don't Know” to the survey question deriving the main outcome variable. The final analytic sample included 137,221 self-respondents who were 18 years and older and completed the labor force interview from 2018 to 2019. The current study was exempt from IRB review based on the use of a publicly available anonymized database.

### 2.2 Measures

#### 2.2.1 Dependent variables

The main outcome variable in the current study, the usual cigar-type, was operationalized as the cigar type used most often. It was derived from the question, “During the PAST 30 days, what type of CIGAR did you use MOST OFTEN?” with the possible responses: “Regular,” “Cigarillos,” and “Little filtered cigars.” As the question was only asked of respondents who used large (regular) cigars, cigarillos, or little filtered cigars every day or some days at the time of the survey, an additional category, non-use, was created for those who were not asked this question.

#### 2.2.2 Independent variables

Independent variables included sociodemographic characteristics and other tobacco product use.

Sociodemographic characteristics included age (18–24, 25–34, 35–44, 45–54, or ≥55 years), sex (male or female), race (non-Hispanic White, non-Hispanic Black, Hispanic, or non-Hispanic other), employment status (full time, part time, unemployed, or not in the labor force), educational attainment (some high school or less, high school graduate or GED, some college [no degree] or associate degree, or at least bachelor's degree), income (<$25,000, $25,000–$50,000, or >$50,000), and residential region (west, northeast, midwest, or south). Other tobacco use was defined as ever using other tobacco products even one time (i.e., e-cigarette, hookah or waterpipe, pipes, and smokeless tobacco such as moist snuff, dip, spit, chew tobacco, or snus) or smoking 100 cigarettes in their lifetime and now smoking cigarettes some days or every day (National Cancer Institute, 2020).

### 2.3 Statistical analysis

Sociodemographic characteristics for the four usual cigar types (large cigars, cigarillos, little filtered cigars, and non-use) are reported in Table 1. The weighted relative frequencies (column percentages) and 95% confidence intervals are reported for all categorical variables. Rao–Scott chi-square tests were used to compare the distribution of characteristics between usual cigar-type use. Multinomial logistic regression was used to assess the association between sociodemographic characteristics and usual cigar-type use (large cigars, cigarillos, and little filtered cigars) relative to non-use. Additional analyses were conducted to examine factors associated with using cigarillos or little filtered cigars relative to large cigars, given that cigar use is historically associated with older adults using traditional large cigars (Malone et al., 2001, Yerger et al., 2001). Analyses adjusted for age, sex, race, employment status, income, educational attainment, other tobacco use, and residential region. Adjusted odds ratios (AORs) and 95% confidence intervals (CI) were reported for multinomial logistic regression. In addition, stratified analyses were performed to examine the difference between male and female respondents given the documented gender difference in cigar use (Higgins et al., 2015, Cullen et al., 2011). Additional analyses examined findings among those who reported some day use (as opposed to every day use). Sampling weights were used in all analyses to account for the differential probability of sample selection and nonresponses. Detailed survey design methodology can be found in the CPS technical paper (Cohn et al., 2015). All tests were 2-sided, and p < 0.05 was considered significant. The listwise deletion was used to manage missing data. All analyses were performed using SAS, version 9.4.

**Table 1:** Descriptive statistics by usual cigar-type use.

	Large cigars	Cigarillos	Little filtered cigars	Non-use
N	1467	513	446	134,795
Age				
18–24	8.8 (6.3, 11.3)	15.0 (9.7, 20.4)	13.2 (7.8, 18.5)	9.7 (9.5, 10.0)
25–34	24.1 (21.4, 26.9)	24.1 (19.3, 28.9)	20.6 (15.8, 25.5)	19.9 (19.6, 20.1)
35–44	16.9 (14.6, 19.1)	18.6 (14.6, 22.6)	16.9 (12.6, 21.2)	16.3 (16.0, 16.5)
45–64	36.1 (33.2, 39.1)	32.0 (27.2, 36.9)	40.2 (34.6, 45.8)	33.2 (32.9, 33.5)
65+	14.0 (12.2, 15.8)	10.3 (7.8, 12.9)	9.1 (6.4, 11.9)	21.0 (20.7, 21.2)
Sex				
Male	92.6 (91.0, 94.3)	77.8 (73.4, 82.2)	69.1 (63.7, 74.5)	47.4 (47.1, 47.8)
Female	7.4 (5.7, 9.0)	22.2 (17.8, 26.6)	30.9 (25.5, 36.3)	52.6 (52.2, 52.9)
Race				
Non-Hispanic White	74.9 (71.9, 77.9)	52.3 (46.7, 57.9)	54.5 (48.5, 60.5)	63.0 (62.6, 63.3)
Non-Hispanic Black	10.6 (8.5, 12.6)	30.3 (24.8, 35.7)	28.4 (22.5, 34.3)	11.8 (11.5, 12.0)
Hispanic	10.3 (8.0, 12.6)	12.5 (8.5, 16.6)	11.7 (7.6, 15.8)	16.7 (16.4, 17.0)
Non-Hispanic Other	4.3 (2.9, 5.7)	4.8 (2.7, 7.0)	5.4 (2.5, 8.3)	8.5 (8.3, 8.8)
Employment status				
Full time	68.0 (65.2, 70.9)	55.5 (50.0, 61.0)	46.3 (40.5, 52.2)	51.9 (51.5, 52.2)
Part time	6.7 (5.2, 8.3)	10.8 (7.2, 14.4)	10.8 (7.2, 14.5)	10.9 (10.7, 11.1)
Unemployed	3.5 (2.3, 4.6)	8.3 (4.4, 12.3)	5.4 (1.8, 8.9)	2.8 (2.6, 2.9)
Not in labor force	21.8 (19.3, 24.2)	25.4 (21.1, 29.6)	37.4 (31.8, 43.1)	34.5 (34.1, 34.8)
Income				
<$25,000	13.4 (11.2, 15.6)	31.5 (26.2, 36.8)	38.8 (33.1, 44.5)	18.3 (18.0, 18.5)
$25,000–$50,000	17.3 (14.9, 19.6)	23.1 (18.4, 27.7)	24.6 (19.6, 29.5)	23.6 (23.3, 23.8)
>$50,000	69.3 (66.4, 72.2)	45.5 (40.0, 51.0)	36.7 (30.9, 42.5)	58.2 (57.8, 58.5)
Educational attainment				
Some high school or less	7.1 (5.3, 8.9)	13.4 (8.8, 18.1)	16.1 (11.8, 20.4)	9.7 (9.5, 9.9)
High school graduate or GED	21.3 (18.7, 23.8)	33.8 (28.6, 38.9)	39.8 (34.1, 45.5)	27.0 (26.7, 27.3)
Some college or Associate degree	32.3 (29.2, 35.3)	36.1 (30.8, 41.5)	30.8 (25.2, 36.5)	29.3 (28.9, 29.6)
At least bachelor's degree	39.4 (36.3, 42.4)	16.7 (13.0, 20.3)	13.3 (9.5, 17.0)	34.0 (33.7, 34.3)
Other tobacco use				
Yes	54.8 (51.7, 57.9)	58.5 (53.0, 63.9)	53.5 (47.6, 59.4)	16.3 (16.1, 16.6)
No	45.2 (42.1, 48.3)	41.5 (36.1, 47.0)	46.5 (40.6, 52.4)	83.7 (83.4, 83.9)
Region				
Northeast	18.6 (16.2, 21.0)	13.0 (9.1, 16.9)	16.6 (11.8, 21.4)	17.5 (17.3, 17.8)
Midwest	24.9 (22.2, 27.5)	29.5 (24.5, 34.6)	17.6 (13.4, 21.8)	20.6 (20.4, 20.9)
South	36.7 (33.6, 39.7)	40.1 (34.7, 45.5)	51.6 (45.8, 57.5)	37.9 (37.6, 38.2)
West	19.9 (17.4, 22.3)	17.4 (13.1, 21.6)	14.2 (10.4, 17.9)	23.9 (23.7, 24.2)

The weighted frequency and its 95% confidence interval were reported for all categorical variables. The distribution differed significantly across four cigar type groups for each sociodemographic characteristic and other tobacco product use (p < 0.001).

## 3 Results

Among the 137,221 adults included in the study, 1467 (1.1%) used large cigars most often during the past 30 days before the survey, 513 (0.4%) used cigarillos most often, 446 (0.3%) used little filtered cigars most often, and the remaining 134,795 (98.1%) did not use large cigars, cigarillos, or little filtered cigars every day or some days at the time of the survey (see supplemental Table 1). Among those who usually used large cigars, about 92.6% were male, 74.9% were non-Hispanic White, and approximately 8.8% were aged 18–24 years. In contrast, among those who usually used cigarillos, 77.8% were male, 52.3% were non-Hispanic White, 30.3% were non-Hispanic Black, and 15.0% were aged 18–24. Among those who usually used filtered cigars, 69.1% were male, 54.5% were non-Hispanic White, 28.4% were non-Hispanic Black, and 13.2% were 18–24 (Table 1). Among those who did not use cigars, 47.4% were male, 63.0% were non-Hispanic White, and 9.7% were 18–24. Sociodemographic characteristics differed significantly across the four cigar type groups. Additionally, over half of those who used cigars reported use of other tobacco products.

The multinomial logistic regression results on usual cigar-type use (large cigars, cigarillos, and little filtered cigars) with non-use as the base category are presented in Table 2. Results are adjusted for age, sex, race, employment status, income, educational attainment, other tobacco use, and residential region. Relative to non-use, we found males had significantly higher odds than females of using large cigars (AOR, 10.30, 95% CI, 8.04–13.19), cigarillos (AOR, 2.92, 95% CI, 2.24–3.80), and little filtered cigars (AOR, 2.02, 95% CI, 1.55–2.64). Likewise, those who used other tobacco products were more likely to use all cigar types. In addition, those living in the midwest region had significantly higher odds of using large cigars (AOR, 1.27, 95% CI, 1.04–1.53) and cigarillos (AOR, 1.69, 95% CI, 1.19–2.41), whereas those living in the south region had significantly higher odds of using little filtered cigars (AOR, 1.81, 95% CI, 1.30–2.52) compared to those living in the west. However, Hispanic, non-Hispanic other, and those not in the labor force had significantly lower odds of using large cigars relative to non-users compared to their reference category (Table 2). Those who had a family income of less than $25,000 and did not obtain a bachelor's degree had higher odds of preferring cigarillos or little filtered cigars relative to non-users. Likewise, compared to non-Hispanic White adults, non-Hispanic Black adults had higher odds of using cigarillos (AOR, 3.95, 95% CI, 2.96–5.36) or little filtered cigars (AOR, 2.75, 95% CI, 2.00–3.78). Similar results were found in the analysis stratified by sex and among those who used cigars some days (see supplemental Tables 2 and 3).

**Table 2:** Multinomial logistic regression on usual cigar-type use relative to non-use, N = 137,221.

	Large cigars vs. non-use	Cigarillos vs. non-use	Little filtered cigars vs. non-use
Age			
18–24	Ref	Ref	Ref
25–34	1.05 (0.74, 1.48)	0.87 (0.54, 1.39)	0.98 (0.58, 1.67)
35–44	0.97 (0.68, 1.37)	0.98 (0.61, 1.56)	1.13 (0.66, 1.95)
45–64	1.09 (0.78, 1.51)	0.90 (0.58, 1.38)	1.25 (0.76, 2.04)
65+	0.75 (0.52, 1.07)	0.50 (0.31, 0.79)	0.36 (0.20, 0.63)
Sex			
Male	10.30 (8.04, 13.19)	2.92 (2.24, 3.80)	2.02 (1.55, 2.64)
Female	Ref	Ref	Ref
Race			
Non-Hispanic White	Ref	Ref	Ref
Non-Hispanic Black	1.12 (0.89, 1.40)	3.95 (2.96, 5.26)	2.75 (2.00, 3.78)
Hispanic	0.71 (0.55, 0.93)	1.09 (0.71, 1.66)	0.85 (0.54, 1.32)
Non-Hispanic Other	0.50 (0.35, 0.72)	0.97 (0.60, 1.57)	1.01 (0.56, 1.83)
Employment status			
Full time	Ref	Ref	Ref
Part time	0.77 (0.59, 1.01)	1.03 (0.68, 1.55)	1.18 (0.77, 1.81)
Unemployed	1.09 (0.76, 1.56)	1.77 (1.07, 2.95)	1.32 (0.65, 2.69)
Not in labor force	0.82 (0.66, 1.00)	0.81 (0.60, 1.11)	1.36 (1.02, 1.80)
Income			
<$25,000	0.91 (0.73, 1.13)	1.79 (1.35, 2.38)	2.31 (1.66, 3.20)
$25,000–50,000	0.77 (0.64, 0.92)	1.10 (0.82, 1.47)	1.37 (0.99, 1.91)
>$50,000	Ref	Ref	Ref
Educational attainment			
Some high school or less	0.84 (0.62, 1.15)	2.32 (1.47, 3.65)	2.80 (1.76, 4.45)
High school graduate or GED	0.71 (0.59, 0.85)	1.93 (1.40, 2.65)	2.58 (1.79, 3.72)
Some college or Associate degree	0.99 (0.84, 1.15)	1.97 (1.44, 2.68)	2.00 (1.37, 2.92)
At least bachelor's degree	Ref	Ref	Ref
Other tobacco use			
Yes	4.03 (3.53, 4.61)	6.66 (5.22, 8.51)	5.85 (4.48, 7.64)
No	Ref	Ref	Ref
Region			
West	Ref	Ref	Ref
Northeast	1.23 (1.00, 1.51)	1.01 (0.65, 1.58)	1.51 (0.97, 2.33)
Midwest	1.27 (1.04, 1.53)	1.69 (1.19, 2.41)	1.18 (0.80, 1.72)
South	1.16 (0.96, 1.39)	1.17 (0.84, 1.65)	1.81 (1.30, 2.52)

Multinomial logistic regression was used to assess the association between sociodemographic characteristics and usual cigar-type use (large cigars, cigarillos, and little filtered cigars) relative to non-use adjusting for age, sex, race, employment status, income, educational attainment, other tobacco use, and residential region. Adjusted odds ratios (AORs) and 95% C.I.s were reported.

Table 3 shows the multinomial logistic regression results for usual cigar-type use with large cigar users as the base category. Relative to those who used large cigars, males had significantly lower odds than females of cigarillo or little filtered cigar use (e.g., cigarillos: AOR, 0.28, 95% CI, 0.20–0.41; little filtered cigars: AOR, 0.20, 95% CI, 0.14–0.28). In contrast, non-Hispanic Black adults (e.g., cigarillos: AOR, 3.54, 95% CI, 2.46–5.07; little filtered cigars: AOR, 2.46, 95% CI, 1.67–3.63), non-Hispanic other adults, those with a family income less than $50,000, and those without a bachelor's degree, and those who used other tobacco products had significantly higher odds of cigarillos or little filtered cigars use relative to large cigars compared to their reference category (Table 3). Results from the stratified analysis by sex are presented in Table 4. Among males, the results were consistent with the findings among the full sample. However, among females, much of the characteristics associated with cigarillos or little filtered cigars use relative to large cigars were not.

**Table 3:** Multinomial logistic regression on usual cigar-type use, with large cigar use as the referent group, N = 137,221.

	Cigarillos vs. Large Cigars	Little filtered cigars vs. Large Cigars
Age		
18–24	Ref	Ref
25–34	0.83 (0.47, 1.49)	0.94 (0.50, 1.76)
35–44	1.01 (0.56, 1.81)	1.17 (0.62, 2.23)
45–64	0.82 (0.48, 1.41)	1.15 (0.64, 2.06)
65+	0.66 (0.37, 1.19)	0.48 (0.25, 0.94)
Sex		
Male	0.28 (0.20, 0.41)	0.20 (0.14, 0.28)
Female	Ref	Ref
Race		
Non-Hispanic White	Ref	Ref
Non-Hispanic Black	3.54 (2.46, 5.07)	2.46 (1.67, 3.63)
Hispanic	1.53 (0.93, 2.52)	1.19 (0.71, 1.99)
Non-Hispanic Other	1.95 (1.06, 3.56)	2.03 (1.01, 4.08)
Employment status		
Full time	Ref	Ref
Part time	1.34 (0.82, 2.17)	1.53 (0.93, 2.53)
Unemployed	1.63 (0.88, 3.01)	1.21 (0.55, 2.69)
Not in labor force	1.00 (0.69, 1.45)	1.66 (1.17, 2.35)
Income		
<$25,000	1.97 (1.38, 2.81)	2.54 (1.72, 3.76)
$25,000–50,000	1.43 (1.02, 2.01)	1.78 (1.23, 2.59)
>$50,000	Ref	Ref
Educational attainment		
Some high school or less	2.75 (1.59, 4.76)	3.33 (1.91, 5.81)
High school graduate or GED	2.73 (1.90, 3.93)	3.66 (2.44, 5.49)
Some college or Associate degree	2.00 (1.42, 2.81)	2.03 (1.35, 3.05)
At least bachelor's degree	Ref	Ref
Other tobacco use		
Yes	1.65 (1.25, 2.18)	1.45 (1.08, 1.95)
No	Ref	Ref
Region		
West	Ref	Ref
Northeast	0.83 (0.51, 1.35)	1.23 (0.76, 1.98)
Midwest	1.34 (0.89, 1.99)	0.93 (0.61, 1.43)
South	1.01 (0.69, 1.49)	1.56 (1.07, 2.28)

Multinomial logistic regression was used to assess the factors associated with the usual cigarillos and little filtered cigars use relative to large cigars adjusting for age, sex, race, employment status, income, educational attainment, other tobacco use, and residential region.

Adjusted odds ratios (AORs) and 95% C.I.s were reported.

**Table 4:** Multinomial logistic regression on usual cigar-type use with large cigars as the referent group, stratified by sex.

	Cigarillos vs. Large cigars		Little filtered cigars vs. Large cigars	
	Male	Female	Male	Female
Age				
18–24	Ref	Ref	Ref	Ref
25–34	0.63 (0.32, 1.21)	1.23 (0.29, 5.23)	1.03 (0.50, 2.15)	0.52 (0.11, 2.41)
35–44	1.06 (0.55, 2.03)	0.54 (0.12, 2.37)	1.46 (0.70, 3.04)	0.44 (0.09, 2.10)
45–64	0.82 (0.45, 1.50)	0.62 (0.16, 2.49)	1.38 (0.73, 2.63)	0.55 (0.12, 2.47)
65+	0.70 (0.37, 1.33)	0.83 (0.15, 4.68)	0.55 (0.27, 1.12)	0.50 (0.08, 3.26)
Race				
Non-Hispanic White	Ref	Ref	Ref	Ref
Non-Hispanic Black	3.96 (2.63, 5.96)	1.49 (0.66, 3.37)	2.63 (1.64, 4.20)	1.07 (0.49, 2.35)
Hispanic	1.59 (0.88, 2.86)	0.75 (0.27, 2.07)	1.59 (0.89, 2.86)	0.26 (0.08, 0.86)
Non-Hispanic Other	1.78 (0.87, 3.64)	1.86 (0.57, 6.05)	1.87 (0.77, 4.52)	1.65 (0.47, 5.82)
Employment status				
Full time	Ref	Ref	Ref	Ref
Part time	1.16 (0.63, 2.11)	1.76 (0.64, 4.80)	1.70 (0.92, 3.13)	1.42 (0.51, 3.95)
Unemployed	2.08 (1.08, 4.01)	0.31 (0.07, 1.39)	1.49 (0.55, 4.01)	0.57 (0.16, 1.98)
Not in labor force	0.92 (0.60, 1.41)	1.45 (0.65, 3.21)	1.86 (1.22, 2.82)	1.78 (0.83, 3.84)
Income				
<$25,000	1.99 (1.33, 2.97)	2.11 (0.99, 4.53)	2.24 (1.42, 3.55)	3.37 (1.60, 7.10)
$25,000–$50,000	1.44 (0.98, 2.09)	1.92 (0.80, 4.58)	1.46 (0.94, 2.25)	3.91 (1.62, 9.41)
>$50,000	Ref	Ref	Ref	Ref
Educational attainment				
Some high school or less	3.10 (1.68, 5.72)	1.07 (0.33, 3.43)	3.86 (2.03, 7.37)	1.34 (0.43, 4.14)
High school graduate or GED	2.72 (1.83, 4.04)	2.40 (0.89, 6.51)	4.21 (2.66, 6.68)	2.14 (0.81, 5.65)
Some college or Associate degree	1.81 (1.23, 2.65)	3.81 (1.53, 9.48)	1.78 (1.10, 2.90)	3.59 (1.43, 9.01)
At least bachelor's degree	Ref	Ref	Ref	Ref
Other tobacco use				
Yes	1.50 (1.10, 2.05)	1.69 (0.86, 3.30)	1.33 (0.93, 1.89)	1.26 (0.66, 2.41)
No	Ref	Ref	Ref	Ref
Region				
West	Ref	Ref	Ref	Ref
Northeast	0.82 (0.47, 1.42)	0.75 (0.23, 2.40)	1.11 (0.66, 1.88)	1.34 (0.43, 4.17)
Midwest	1.31 (0.84, 2.04)	1.45 (0.50, 4.24)	0.94 (0.57, 1.54)	0.91 (0.32, 2.56)
South	1.04 (0.68, 1.59)	0.69 (0.27, 1.79)	1.56 (1.00, 2.44)	1.14 (0.50, 2.59)

Multinomial logistic regression was used to assess the factors associated with the usual cigarillos and little filtered cigars use relative to large cigars, adjusting for age, race, employment status, income, educational attainment, other tobacco use, and residential region.

Adjusted odds ratios (AORs) and 95% C.I.s were reported.

## 4 Discussion

This 2018–2019 TUS-CPS data analysis identified significant sociodemographic differences based on usual cigar-type use. Characteristics of those who usually used large cigars differed significantly from those who usually used cigarillos, little filtered cigars, or reported no cigar use. These findings extend previous work in this area and have important implications for practice.

We identified differences in usual cigar-type use by race, income, education, and other product use. Non-Hispanic Black adults were more likely to use all cigar products and then more likely to prefer cigarillos and little filtered cigars than large cigars compared with non-Hispanic White adults. Multiple studies have reported higher rates of cigarillo and little filtered cigar use among Black adults (Corey et al., 2014, Borawski et al., 2010, Chen-Sankey et al., 2021). As noted previously (Weinberger et al., 2002), while cigar use rates among non-Hispanic White adults and Hispanic adults have declined, cigar use rates among Black adults have not. Non-White communities have been targeted by the tobacco industry, with more advertisements and lower prices which likely helps account for increases in use among these populations (Cantrell et al., 2013, Smiley et al., 2019). We found that Hispanic adults and adults of other races had lower odds than White adults for large cigar use relative to non-use. This is similar to another study that found that the prevalence was lowest among adults of other non-Hisparaces compared to other racial groups regardless of cigar type (Chen-Sankey et al., 2021). In the present study, the non-Hispanic other category was predominantly Asian, though lower use rates prevented us from establishing a standalone category. However, the findings of this group suggest that research with larger samples to allow parsing out this population might be warranted.

This study also found that those with higher income and educational attainment were less likely to use cigarillos and filtered cigars, which echoes previous findings and suggests low-income individuals remain a priority population (Corey et al., 2018, Borawski et al., 2010, Corey et al., 2014). Increasing price and minimum pack sizes may effectively reduce use among this population (King et al., 2020, Persoskie et al., 2019). These findings also highlight that it might be beneficial to target prevention efforts to particular subgroups, given that cultural values and socioeconomic status may influence cigar use (Nguyen, 2019, Srinivasan and Guillermo, 2000). Lastly, other tobacco use was common among those reporting cigar use and increased the odds of all types of cigar use. However, it was even more pronounced among those who usually use cigarillos and little filtered cigars. This corresponds to other literature that has found some little cigars to be used as substitutes for cigarettes (Corey et al., 2018, Delnevo et al., 2017). Effective messaging on the harms of poly tobacco use is needed.

Males had greater odds of using all cigar products but lower odds than females of using cigarillos and filtered cigars relative large cigars. This is similar to another study that found that females were more likely to use cigarillos or little cigars (Richardson et al., 2013). We extended this research by stratifying analyses based on sex. While most findings remained for males, we saw few significant results among females. This may reflect the smaller sample size of female cigar users, and, as such, the overall results are likely driven by males. Further research is needed among larger sample sizes of female cigar users to identify whether there are unique risk factors for use in this population, and, if so, inform tailored messaging.

Concerning age, we found lower odds for the use of cigarillos and little filtered cigars among those aged at least 65 years old compared with 18–24-year-olds, but no differences among other age groups or for other products. This contrasts findings from other studies that found that 18–24-year-olds are more likely to report cigarillo use than large cigar use (Corey et al., 2018, Borawski et al., 2010, Richardson et al., 2013, Corey et al., 2014). It is unclear why these differences were not identified in the present study. Possible reasons include the population shifting to higher age categories as these data are more recent than previous studies. Also, it is unclear to what extent the results are due to the changing tobacco product and policy landscapes (Delnevo et al., 2017, King, 2020) which may influence cigar appeal across age groups. The findings underscore the importance of not solely focusing on young adults with prevention, cessation, and policy efforts. For example, while systematically assessing cigar use in clinical settings is underutilized (LeLaurin et al., 2021, Polubriaginof et al., 2018), encouraging clinicians to prioritize all age groups instead of focusing on those historically thought more likely to smoke cigars is important.

There are several limitations to consider concerning these findings. First, TUS-CPS is a self-reported survey, which may result in recall bias. Second, the analysis did not assess blunt and premium cigar use because this information was not available in the survey. In addition, there is a possibility that respondents may misunderstand the different cigar classifications. However, a detailed description and common brands for the different cigar types were provided during the survey. Lastly, the results make no claim on causality based on the analytical design. Notwithstanding the limitations, the present study extends the limited research on factors associated with usual cigar-type use using a nationally representative U.S. sample of adults from the TUS-CPS.

## 5 Conclusions

Cigars lack the policy, prevention, and cessation efforts to reduce use, despite similar risks compared to combustible cigarettes. The findings from this nationally representative analysis of 2018–2019 TUS-CPS highlight potential areas for targeted efforts, including education on the risks of polytobacco use, broadening prevention and clinician assessment efforts across age groups, and continued efforts to implement policies that reduce the disproportionate rates of use among minoritized and low-income populations.

## Disclosure of funding

Jessica King acknowledges research support by NCI and FDA 10.13039/100010628Center for Tobacco Products (CTP), grant number K01CA253235. The content is solely the responsibility of the authors and does not necessarily represent the official views of the NIH or the Food and Drug Administration.

## CRediT authorship contribution statement

**Sunday Azagba:** Conceptualization, Investigation, Methodology, Project administration, Supervision, Writing - original draft, Writing - review & editing. **Jessica L. King:** Funding, Writing - original draft, Writing - review & editing. **Lingpeng Shan:** Formal analysis, Writing - review & editing.

## Declaration of Competing Interest

The authors declare that they have no known competing financial interests or personal relationships that could have appeared to influence the work reported in this paper.
