# Supplementary material for: Characteristics differ based on usual cigar-type use among U.S. adults: Analysis from the tobacco use supplement to the current population survey
Source: Prev Med Rep. 2021 Sep 15;24:101560. doi: 10.1016/j.pmedr.2021.101560 (PMC8683945; doi:10.1016/j.pmedr.2021.101560)
Supplement: Supplementary data 1 [file mmc1.docx]

**Supplementary Table 1: Descriptive statistics for full sample (n= 137,221)**

|  | Full Sample |
| --- | --- |
| Age |  |
| 18-24 | 9.76 (9.48, 10.04) |
| 25-34 | 19.95 (19.67, 20.23) |
| 35-44 | 16.29 (16.06, 16.53) |
| 45-64 | 33.21 (32.90, 33.51) |
| 65+ | 20.79 (20.56, 21.02) |
| Sex |  |
| Male | 48.19 (47.86, 48.53) |
| Female | 51.81 (51.47, 52.14) |
| Race |  |
| Non-Hispanic White | 63.07 (62.73, 63.41) |
| Non-Hispanic Black | 11.86 (11.62, 12.10) |
| Hispanic | 16.60 (16.32, 16.88) |
| Non-Hispanic Other | 8.47 (8.27, 8.68) |
| Employment status |  |
| Full time | 52.10 (51.77, 52.44) |
| Part time | 10.83 (10.61, 11.06) |
| Unemployed | 2.81 (2.69, 2.93) |
| Not in labor force | 34.25 (33.94, 34.56) |
| Income |  |
| <$25,000 | 18.35 (18.09, 18.60) |
| $25,000-$50,000 | 23.47 (23.19, 23.76) |
| >$50,000 | 58.18 (57.85, 58.51) |
| Educational attainment |  |
| Some high school or less | 9.72 (9.51, 9.94) |
| High school graduate or GED | 27.02 (26.72, 27.33) |
| Some college or Associate degree | 29.33 (29.01, 29.64) |
| At least bachelor's degree | 33.93 (33.61, 34.24) |
| Other tobacco use |  |
| Yes | 17.15 (16.90, 17.40) |
| No | 82.85 (82.60, 83.10) |
| Region |  |
| West | 17.53 (17.26, 17.79) |
| Northeast | 20.71 (20.44, 20.97) |
| Midwest | 37.93 (37.61, 38.26) |
| South | 23.83 (23.55, 24.11) |
| Cigar Preference |  |
| Large cigars | 1.11 (1.04, 1.18) |
| Cigarillos | 0.41 (0.36, 0.45) |
| Little filtered cigars | 0.34 (0.30, 0.38) |
| Non-use | 98.14 (98.05, 98.23) |

The weighted frequency and its 95% confidence interval were reported for all categorical variables.

**Supplementary Table 2**: Multinomial logistic regression on usual cigar-type relative to non-use, stratified by sex

|  | | Large cigars vs. non-use | | Cigarillos vs. non-use | | | Little filtered cigars vs. non-use | | |
| --- | --- | --- | --- | --- | --- | --- | --- | --- | --- |
|  | Male | | Female | | Male | Female | | Male | Female |
| Age |  | |  | |  |  | |  |  |
| 18-24 | Ref | | Ref | | Ref | Ref | | Ref | Ref |
| 25-34 | 1.00 (0.70, 1.44) | | 1.75 (0.51, 6.03) | | 0.63 (0.36, 1.10) | 2.15 (1.00, 4.62) | | 1.03 (0.55, 1.96) | 0.90 (0.36, 2.28) |
| 35-44 | 0.91 (0.63, 1.32) | | 1.87 (0.56, 6.28) | | 0.96 (0.56, 1.66) | 1.01 (0.43, 2.39) | | 1.33 (0.70, 2.52) | 0.83 (0.31, 2.22) |
| 45-64 | 1.05 (0.75, 1.48) | | 1.69 (0.53, 5.39) | | 0.86 (0.52, 1.43) | 1.05 (0.49, 2.25) | | 1.45 (0.84, 2.52) | 0.93 (0.36, 2.41) |
| 65+ | 0.74 (0.51, 1.07) | | 0.59 (0.14, 2.46) | | 0.52 (0.31, 0.87) | 0.49 (0.18, 1.30) | | 0.41 (0.22, 0.75) | 0.29 (0.09, 0.99) |
| Race |  | |  | |  |  | |  |  |
| Non-Hispanic White | Ref | | Ref | | Ref | Ref | | Ref | Ref |
| Non-Hispanic Black | 1.00 (0.78, 1.28) | | 2.63 (1.43, 4.82) | | 3.95 (2.83, 5.53) | 3.92 (2.25, 6.80) | | 2.63 (1.75, 3.93) | 2.80 (1.69, 4.66) |
| Hispanic | 0.65 (0.49, 0.86) | | 1.78 (0.83, 3.80) | | 1.03 (0.61, 1.72) | 1.33 (0.67, 2.64) | | 1.03 (0.62, 1.72) | 0.47 (0.19, 1.16) |
| Non-Hispanic Other | 0.48 (0.33, 0.71) | | 0.75 (0.31, 1.81) | | 0.86 (0.47, 1.56) | 1.40 (0.64, 3.06) | | 0.90 (0.41, 1.99) | 1.24 (0.50, 3.05) |
| Employment status |  | |  | |  |  | |  |  |
| Full time | Ref | | Ref | | Ref | Ref | | Ref | Ref |
| Part time | 0.78 (0.59, 1.04) | | 0.68 (0.31, 1.48) | | 0.91 (0.53, 1.55) | 1.19 (0.63, 2.24) | | 1.33 (0.77, 2.30) | 0.96 (0.50, 1.86) |
| Unemployed | 1.01 (0.69, 1.49) | | 1.85 (0.77, 4.48) | | 2.10 (1.22, 3.63) | 0.58 (0.17, 1.95) | | 1.50 (0.60, 3.75) | 1.05 (0.43, 2.59) |
| Not in labor force | 0.84 (0.67, 1.04) | | 0.62 (0.34, 1.13) | | 0.77 (0.53, 1.12) | 0.89 (0.53, 1.50) | | 1.55 (1.09, 2.22) | 1.10 (0.68, 1.77) |
| Income |  | |  | |  |  | |  |  |
| <$25,000 | 0.89 (0.70, 1.13) | | 0.93 (0.57, 1.51) | | 1.76 (1.27, 2.45) | 1.96 (1.08, 3.53) | | 1.99 (1.34, 2.96) | 3.12 (1.77, 5.50) |
| $25,000-$50,000 | 0.80 (0.66, 0.96) | | 0.49 (0.26, 0.93) | | 1.14 (0.82, 1.59) | 0.95 (0.52, 1.72) | | 1.16 (0.78, 1.73) | 1.93 (1.05, 3.54) |
| >$50,000 | Ref | | Ref | | Ref | Ref | | Ref | Ref |
| Education attainment |  | |  | |  |  | |  |  |
| Some high school or less | 0.77 (0.55, 1.09) | | 1.79 (0.84, 3.83) | | 2.40 (1.44, 4.00) | 1.91 (0.78, 4.65) | | 2.99 (1.72, 5.18) | 2.39 (1.03, 5.56) |
| High school graduate or GED | 0.70 (0.58, 0.85) | | 0.85 (0.43, 1.65) | | 1.91 (1.34, 2.72) | 2.03 (0.97, 4.26) | | 2.96 (1.94, 4.52) | 1.81 (0.90, 3.66) |
| Some college or Associate degree | 1.02 (0.87, 1.20) | | 0.63 (0.33, 1.18) | | 1.84 (1.30, 2.61) | 2.39 (1.24, 4.62) | | 1.82 (1.14, 2.88) | 2.25 (1.16, 4.40) |
| At least bachelor's degree | Ref | | Ref | | Ref | Ref | | Ref | Ref |
| Other tobacco use |  | |  | |  |  | |  |  |
| Yes | 3.86 (3.37, 4.43) | | 6.05 (3.70, 9.87) | | 5.79 (4.37, 7.67) | 10.21 (6.45, 16.15) | | 5.12 (3.69, 7.10) | 7.63 (4.98, 11.69) |
| No | Ref | | Ref | | Ref | Ref | | Ref | Ref |
| Region |  | |  | |  |  | |  |  |
| West | Ref | | Ref | | Ref | Ref | | Ref | Ref |
| Northeast | 1.21 (0.98, 1.51) | | 1.43 (0.67, 3.03) | | 0.99 (0.60, 1.65) | 1.06 (0.44, 2.60) | | 1.35 (0.84, 2.18) | 1.91 (0.81, 4.49) |
| Midwest | 1.26 (1.03, 1.54) | | 1.30 (0.59, 2.88) | | 1.65 (1.10, 2.47) | 1.89 (0.91, 3.90) | | 1.18 (0.75, 1.87) | 1.18 (0.61, 2.31) |
| South | 1.12 (0.92, 1.36) | | 1.78 (0.97, 3.28) | | 1.16 (0.79, 1.70) | 1.23 (0.59, 2.57) | | 1.75 (1.17, 2.62) | 2.02 (1.15, 3.54) |

Multinomial logistic regression was used to assess the factors associated with the preference for cigarillos and little filtered cigars relative to large cigars, adjusting for age, race, employment status, income, educational attainment, other tobacco use, and residential region.

Adjusted odds ratios (AORs) and 95% C.I.s were reported.

**Supplementary Table 3**: Multinomial logistic regression on usual cigar-type relative to non-use among some days cigar users

|  | Cigarillos vs. Large Cigars | Little filtered cigars vs. Large Cigars |
| --- | --- | --- |
| Age |  |  |
| 18-24 | Ref | Ref |
| 25-34 | 0.64 (0.34, 1.19) | 0.62 (0.32, 1.21) |
| 35-44 | 0.68 (0.36, 1.28) | 0.74 (0.37, 1.47) |
| 45-64 | 0.62 (0.35, 1.12) | 0.78 (0.42, 1.44) |
| 65+ | 0.75 (0.38, 1.47) | 0.44 (0.20, 0.93) |
| Sex |  |  |
| Male | 0.29 (0.19, 0.44) | 0.19 (0.12, 0.29) |
| Female | Ref | Ref |
| Race |  |  |
| Non-Hispanic White | Ref | Ref |
| Non-Hispanic Black | 3.67 (2.38, 5.68) | 3.08 (1.95, 4.87) |
| Hispanic | 1.98 (1.10, 3.56) | 1.92 (1.01, 3.65) |
| Non-Hispanic Other | 1.28 (0.64, 2.54) | 1.34 (0.60, 2.99) |
| Employment status |  |  |
| Full time | Ref | Ref |
| Part time | 1.51 (0.87, 2.61) | 1.50 (0.81, 2.77) |
| Unemployed | 1.69 (0.81, 3.55) | 0.86 (0.31, 2.44) |
| Not in labor force | 0.85 (0.53, 1.35) | 1.38 (0.86, 2.22) |
| Income |  |  |
| <$25,000 | 1.84 (1.18, 2.88) | 2.39 (1.45, 3.92) |
| $25,000-$50,000 | 1.43 (0.94, 2.16) | 1.51 (0.96, 2.38) |
| >$50,000 | Ref | Ref |
| Educational attainment |  |  |
| Some high school or less | 2.32 (1.19, 4.52) | 2.42 (1.17, 5.01) |
| High school graduate or GED | 2.90 (1.90, 4.42) | 3.70 (2.25, 6.07) |
| Some college or Associate degree | 1.72 (1.14, 2.58) | 1.88 (1.15, 3.08) |
| At least bachelor's degree | Ref | Ref |
| Other tobacco use |  |  |
| Yes | 1.50 (1.06, 2.11) | 1.31 (0.90, 1.90) |
| No | Ref | Ref |
| Region |  |  |
| West | Ref | Ref |
| Northeast | 1.06 (0.61, 1.83) | 1.52 (0.87, 2.66) |
| Midwest | 1.27 (0.81, 2.01) | 0.94 (0.55, 1.60) |
| South | 0.94 (0.61, 1.46) | 1.35 (0.84, 2.15) |

Multinomial logistic regression was used to assess the association between sociodemographic characteristics and cigar type preference (large cigars, cigarillos, and little filtered cigars) relative to non-use adjusting for age, sex, race, employment status, income, educational attainment, other tobacco use, and residential region. Adjusted odds ratios (AORs) and 95% C.I.s were reported.
